# Topography of Synchronization of Somatosensory Evoked Potentials Elicited by Stimulation of the Sciatic Nerve in Rat

**DOI:** 10.3389/fncom.2016.00043

**Published:** 2016-05-04

**Authors:** Xuefeng Qu, Jiaqing Yan, Xiaoli Li, Peixun Zhang, Xianzeng Liu

**Affiliations:** ^1^Division of the Comprehensive Epilepsy Center and Neurofunctional Monitoring Laboratory, Department of Neurology, Peking University People's HospitalBeijing, China; ^2^School of Electrical and Control Engineering, North China University of TechnologyBeijing, China; ^3^State Key Laboratory of Cognitive Neuroscience and Learning and IDG/McGovern Institute for Brain Research, Beijing Normal UniversityBeijing, China; ^4^Department of Trauma and Orthopaedics, Peking University People's HospitalBeijing, China

**Keywords:** somatosensory evoked potentials, correlation matrix analysis, surrogate resampling, brain functional network, cortical plasticity, peripheral nerve injury, rat

## Abstract

**Purpose:** Traditionally, the topography of somatosensory evoked potentials (SEPs) is generated based on amplitude and latency. However, this operation focuses on the physical morphology and field potential-power, so it suffers from difficulties in performing identification in an objective manner. In this study, measurement of the synchronization of SEPs is proposed as a method to explore brain functional networks as well as the plasticity after peripheral nerve injury.

**Method:** SEPs elicited by unilateral sciatic nerve stimulation in twelve adult male Sprague-Dawley (SD) rats in the normal group were compared with SEPs evoked after unilateral sciatic nerve hemisection in four peripheral nerve injured SD rats. The characterization of synchronized networks from SEPs was conducted using equal-time correlation, correlation matrix analysis, and comparison to randomized surrogate data. Eigenvalues of the correlation matrix were used to identify the clusters of functionally synchronized neuronal activity, and the participation index (PI) was calculated to indicate the involvement of each channel in the cluster. The PI value at the knee point of the PI histogram was used as a threshold to demarcate the cortical boundary.

**Results:** Ten out of the twelve normal rats showed only one synchronized brain network. The remaining two normal rats showed one strong and one weak network. In the peripheral nerve injured group, only one synchronized brain network was found in each rat. In the normal group, all network shapes appear regular and the network is largely contained in the posterior cortex. In the injured group, the network shapes appear irregular, the network extends anteriorly and posteriorly, and the network area is significantly larger. There are considerable individual variations in the shape and location of the network after peripheral nerve injury.

**Conclusion:** The proposed method can detect functional brain networks. Compared to the results of the traditional SEP-morphology-based analysis method, the synchronized functional network area is much larger. Furthermore, the proposed method can also characterize the rapid cortical plasticity after a peripheral nerve is acutely injured.

## Introduction

To map the topography of the rat somatosensory cortex, traditionally, microscopic and histological techniques have been applied. For neuroanatomical tract tracings, horseradish peroxidase (HRP) retrograde tracing method was first introduced and subsequently modified as conjugate with wheat germ agglutinin (Gonatas et al., 1979) or the β-subunit of cholera toxin (Sugimotoa et al., 1997). Fluorescent dyes, which can be used individually or combined with HRP, have served as methods for the mapping of fiber connections. Electrophysiological techniques, such as somatosensory and motor evoked potentials (SEPs and MEPs; Agrawal et al., 2010; Sherman et al., 2010; Cloud et al., 2012; Hua et al., 2012; Pan et al., 2012), have been used to reliably assess the integrity and functionality of sensory pathways and to perform systematic investigation of central or peripheral nervous injury, though with some difficulty in interpretation. More recently, neuroimaging is playing an increasing role in the investigation of the nervous system. In general, histological, electrophysiological, and neuroimaging methods, independently or combined, comprise the toolbox for modern neuroscience research.

SEPs have been widely used in animal experiments on pain (Schaap et al., 2013a,b), spinal cord injury (Sherman et al., 2010; Cloud et al., 2012; Morris et al., 2013; Bazley et al., 2014; Wang et al., 2015a,b), and neuropathy (Niknami et al., 2013; Geis et al., 2015), as well as intraoperative monitoring in clinical settings (Møller, 2010). In the context of spinal cord injury, SEPs have been introduced to assess the extent of damage of the spinal cord (Cloud et al., 2012) and to test the duration of complete signal (SEP) loss that could be tolerated (Morris et al., 2013). Up to now, there have been no consistent standard for SEPs for detecting injury. Generally, a 50% decrease of amplitude or a 10% increase of latency has been used as the alarm threshold value for a nerve injury event (Agrawal et al., 2010). Changes of amplitude and latency over time have been measured to explore cortical plasticity after spinal cord injury (Bazley et al., 2014). Topography of SEPs based on amplitudes (Hollenberg et al., 2006; Hosp et al., 2008) has been delineated. The map is easy to interpret and intuitively displays the somatosensory representation of forelimb and hindlimb, in spite of artificial rules on amplitude thresholds. SEP topography enables further assessment of the somatosensory cortex, studies on learning (Hosp et al., 2008), and monitoring of plasticity progress after spinal cord injury or peripheral nerve injury. Wang et al. put forward time-frequency analysis of SEP signals based on the Matching Pursuit algorithm and short-time Fourier transform and tried to identify the location of neurological impairment in cortex after spinal cord injury (Wang et al., 2015a), but this analysis is still amplitude-based. The above methods either only investigate the threshold for alarm with respect to functional integrity in an isolated functional system or do not explore the plasticity of the cortical network. Sherman et al. tried using the adaptive coherence method to minimize the effect of amplitude and provided an excellent supplement to the traditional use of amplitude for capturing injury-related changes in SEPs after spinal cord injury (Sherman et al., 2010). However, the study did not consider network plasticity.

Synchronization commonly subserves neural networks (Santos-Sierra et al., 2015; Zeng et al., 2015). Thus, determination of synchronized networks may aid in the investigation of cortical network plasticity after peripheral nerve injury. SEPs elicited by unilateral sciatic nerve stimulation were collected from 12 adult Sprague-Dawley (SD) rats, serving as the normal group. SEPs evoked after unilateral sciatic nerve hemisection in four peripheral nerve injured SD rats were collected to study neural changes after injury. Synchronized networks based on SEPs were determined using equal-time correlation, correlation matrix analysis, and comparison to random surrogate data. Eigenvalues of the correlation matrix were used to identify clusters of functionally synchronized neuronal activity, and the participation index (PI) was calculated to indicate the involvement of each channel in the cluster. The PI value at the knee point of the PI histogram was used as a threshold to demarcate the cortical boundary. Finally, the network plasticity after acute peripheral nerve injury was characterized using these methods.

## Materials and methods

### Animals and surgical preparation

Twelve adult male SD rats (250–350 g, obtained from Chinese Academy of Medical Sciences and Peking Union Medical College) comprised the normal group, and four adult male SD rats with unilateral sciatic nerve hemisected comprised the peripheral nerve injured group. Rats were housed in cages with a 12/12 h light/dark cycle, 22 ± 2°C, 50 ± 5% relative humidity, and free access to sufficient food and water. Preoperative fasting for 8 h was enforced, but no water deprivation was enforced. Animal care and surgical procedures were approved by and conducted in accordance with the guidelines of the institutional animal care committee. Craniotomy was performed on the 12 normal rats (six rats on the right side and six rats on the left side) and on the four peripheral nerve injured rats (two rats on each side).

Anesthesia was induced by 50 mg/kg of 5 g/L sodium pentobarbital via intraperitoneal injection. Rats continued to breathe spontaneously and the depth of anesthesia during SEP recording was maintained using 10 mg/kg sodium pentobarbital as needed.

The experiment was performed at 22–26°C room temperature. The rats were placed on a homeothermic blanket system with rectal temperature maintained at 37–37.5°C throughout the surgery and recording. The head region and the hip area were shaved, and rats were fixed in a stereotaxic frame. A midline incision was made. The skull was exposed and craniotomy performed (1 mm medial to 6 mm lateral, 5 mm anterior to 7.5 mm posterior to the bregma) over one hemisphere. Contralateral sciatic nerve was exposed at the inferior border of the ectogluteus without any artificial injury in the normal group. In the peripheral nerve injured group, the peroneal nerve, including the superficial and deep branches, was cut, a 3 mm segment was removed, and then the distal and proximal ends were ligated. However, the tibial nerve component was spared.

### SEP recording

**Figure 1:**
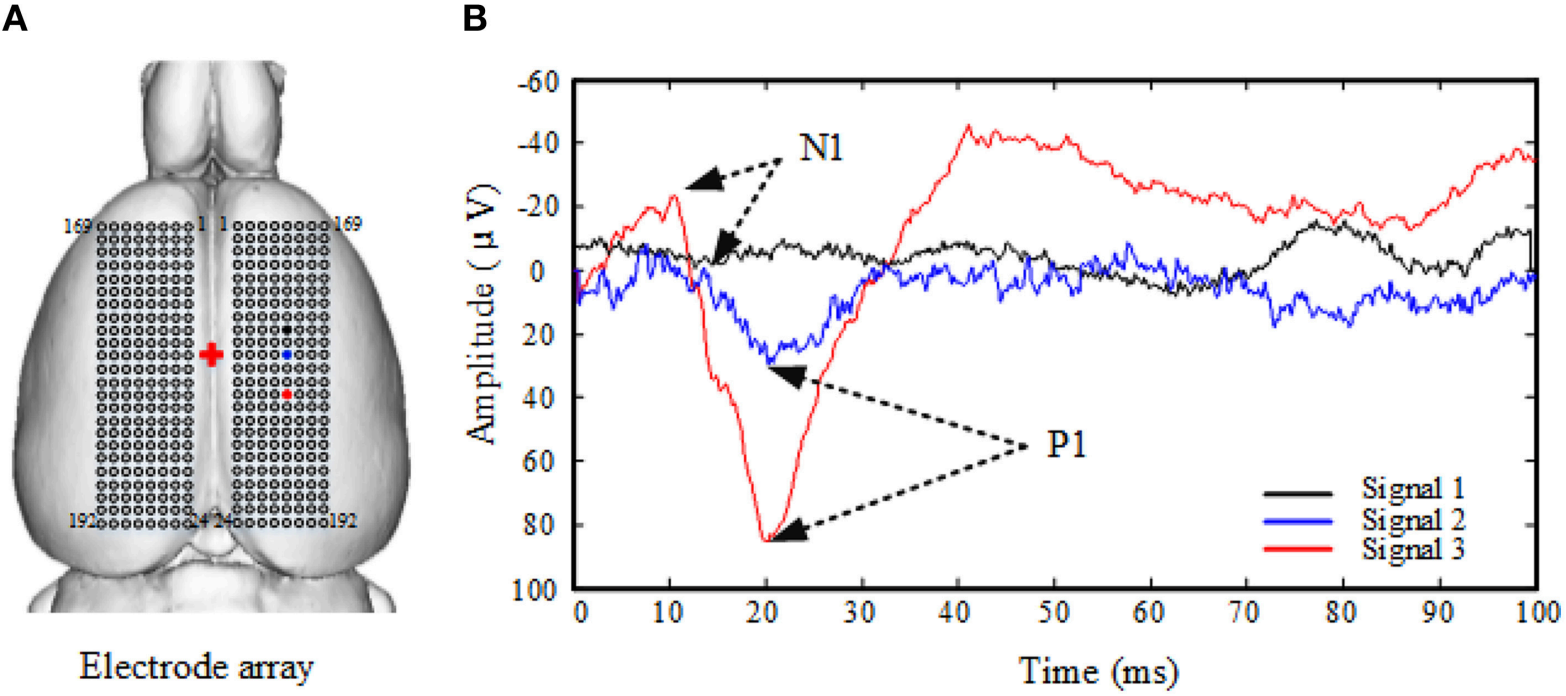
**Electrode array diagram and three example traces of original SEP signals of the sciatic nerve in one normal rat**. **(A)** 192 electrodes record from the left or right side. Numbers indicate channel numbering scheme. The red cross indicates the bregma. The black, blue, and red dots are the recording sites of signals in **(B)**. **(B)** Original SEP signals of the sciatic nerve consisted of a low-amplitude negative wave (N1) followed by a high-amplitude positive wave (P1). Example Signal 1 (black) was obtained from a recording site far away (2.5 mm) from the center of the representative area; signal 2 (blue) was from a site close (1.5 mm) to the center of the representative area; signal 3 (red) was from the center of the representative area. The traces are each averages of 200 sweeps.

SEP traces were collected 30 min after surgery. An electrical stimulator (Neuron-Spectrum-5, Neurosoft, Russia) was used for stimulation of the sciatic nerve via steel bipolar hook electrodes (1 mm diameter) separated by 3 mm distance. A monophasic-square-wave constant current pattern (3 Hz, 100 us pulse width) was used. The stimulation intensity was set as the intensity that made the paw twitch 3 mm. Custom-made, four-channel epidural Ag/AgCl spherical electrodes were used in the acquisition of the cortical signals. The recording sites were separated by 0.5 mm and moved in the medial-lateral and anterior–posterior directions as shown in Figure 1A. Cortical SEPs were amplified by Neuron-Spectrum-5 (gain: 1300, sampling rate: 20 kHz). Two hundred sweeps were averaged for the acquisition of an SEP trace. As shown in Figure 1B, the original SEP signals of the sciatic nerve consisted of a negative wave (N1) followed by a positive, high-amplitude wave (P1). Traditionally, an SEP like signal 2 in Figure 1B is usually ignored when deciding the representation area due to its “small” peak, with amplitude less than 60% of the maximum value (Hosp et al., 2008). This shows a weakness of the traditional amplitude-based processing.

#### Analysis methods

The SEP trace series is denoted *X*_*i*_(*t*_*k*_), *i* = *1…M, M* is the number of channels, and *t*_*k*_ (*k* = 1…*T*) indicates which time sample point, with *T* the number of time points. The data set is normalized as *z*_*i*_(*t*_*k*_):

$$\begin{array}{l} z_{i}\left(t_{k}\right)=\frac{x_{i}\left(t_{k}\right)-<x_{i}>}{\sigma_{i}}, \end{array}$$

where < *X*_*i*_ > and σ_*i*_ are the mean and the standard deviation of *X*_*i*_(*t*_*k*_), respectively. The equal-time correlation method is applied to estimate correlation of the data set and generate a correlation matrix *C*, defined as

$$\begin{array}{l} C_{ij}=\frac{1}{T}\sum_{k}z_{i}\left(t_{k}\right)z_{j}\left(t_{k}\right). \end{array}$$

The eigendecomposition of matrix *C* is performed to compute eigenvalues λ_*i*_,

$$\begin{array}{l} {Cv_{i}=\lambda }_{i}v_{i}, \end{array}$$

where *v*_*i*_ is the eigenvector corresponding to λ_*i*_. The eigenvalues provide information about the synchronization between channels, and the highest eigenvalue may measure the global synchronization.

Subsequently, the eigenvalues of the correlation matrix *C* were tested for significance in order to identify the number of clusters of channels, corresponding to separate functional networks. To do this, we compute the distribution of eigenvalues for the null hypothesis (of no synchrony clusters) by making a surrogate data set in which the phase of the data is randomized. We randomize the phase relationship of all channels of the time series to compute a surrogate correlation matrix *R*; let the eigenvalues of *R* be denoted $\lambda_{m}^{\prime }$ (*m* = 1,…, *M*). We repeat this randomization process *N* times (*N* = 100 for this study) and compute the mean and standard deviation of the eigenvalues as $\bar{\lambda_{i}^{\prime }}$ and *SD*_*i*_, respectively. To randomize the phase relationship of the channels, we use the amplitude-adjusted Fourier transform (AAFT) (Schreiber and Schmitz, 1996), as follows. The linear properties of each channel can be specified by the squared amplitude of its discrete Fourier transform *Y*_*k*_,

$$\begin{array}{l} {\left|Y_{k}\right|}^{2}={\left|\frac{1}{\sqrt{T}}\sum_{t=0}^{T-1}x\left(t\right)\exp\left(\frac{i2\pi kt}{T}\right)\right|}^{2}. \end{array}$$

The surrogate time series *x*′ can be created by multiplying the Fourier transform of the original channels by random phases and then computing the inverse Fourier transform to return to the time domain:

$$\begin{array}{l} x^{\prime }=\frac{1}{\sqrt{T}}{\left|\overset{T-1}{\underset{t=0}{\sum }}Y_{k}e^{i\theta_{k}}\text{exp}\left(\frac{-i2\pi \text{kt}}{\text{T}}\right)\right|}^{2}, \end{array}$$

where 0 ≤ θ_*k*_ ≤ 2π are independent uniform random numbers. Thus, the surrogate series shares the same power spectrum as the original one, but the phase is randomized.

The number of eigenvalues of *C* larger than the confidence interval computed from the null distribution for eigenvalues indicates the number of clusters (i.e., separate functional networks). When the eigenvalue λ_*i*_ is more than K standard deviations larger than the mean surrogate eigenvalue (i.e., $\lambda_{i}>\bar{\lambda_{i}^{\prime }}+K\times {SD}_{i}$, where K is a constant that determines the significance threshold), this suggests the presence of a cluster which include at least two elements of the correlation matrix. Using the 3σ law for a Gaussian distribution, we take the number of eigenvalues exceeding the 99.7% confidence interval (i.e., *K* = 3) as the number of clusters of locally synchronized activity.

We next define an index on the strength of a channel's participation in the functional network, and use this index to determine the extent of the network. Corresponding to the eigenvalues, the eigenvectors characterize the involvement of each channel in a cluster. Let *v*_*ji*_ be the *j*th element of eigenvector *v*_*i*_ with λ_*i*_ as its corresponding eigenvalue. Then, $v_{ji}^{2}$ shows the weight value of channel *j* in cluster *i*. Thus, which channel belongs to which cluster can be determined by the eigenvalues coupled with the eigenvectors. We define the *participation index* (PI) as

$$\begin{array}{l} PI_{ji}=\lambda_{i}v_{ji}^{2}. \end{array}$$

We can then derive a cortical topography from this PI.

Using the level set segmentation method (Cremers et al., 2007), if a threshold is set on PI, the cortical locations corresponding to the level set correspond to a boundary for the putative functional network. We take the boundary with minimum gradient with respect to the threshold as the boundary demarcating the main somatosensory network.

To detect the boundary with minimum gradient, we decrease the PI threshold gradually, which results in a gradual increase in the number of the channels within the boundary. When the increase in the number of channels slows, the current boundary has reached the minimum gradient. This PI threshold corresponds to the knee point on the plot of the PI histogram. Then, the current boundary is taken as the extent of the SEP-induced functional network.

### Statistical analysis

The two-tailed independent student's *t*-test was used to test for significance, with *P* < 0.05 regarded as statistically significant.

## Results

The stimulation intensity to induce SEPs for the normal group and the peripheral nerve injured group was 0.5–0.8 mA and 1.2–2.2 mA, respectively. The original SEP signals of the sciatic nerve was consisted of a low-amplitude negative wave (N1) followed by a high-amplitude positive wave (P1) (Figure 1B). SEP signals of the normal group had longer N1 latency (normal: 9.48 ± 1.51 ms vs. peripheral nerve injured: 7.58 ± 0.75 ms, *P* < 0.05) and longer P1 latency (normal: 20.93 ± 2.12 ms vs. peripheral nerve injured: 18.52 ± 3.23 ms, *P* < 0.05), but smaller N1-P1 amplitude (normal: 49.42 ± 20.74 μV vs. peripheral nerve injured: 93.63 ± 13.61 μV, *P* < 0.05). No significant difference in N1-P1 interval was found between the two groups (normal: 11.44 ± 1.18 ms vs. peripheral nerve injured: 10.94 ± 2.65 ms, *P* = 0.175).

**Figure 2:**
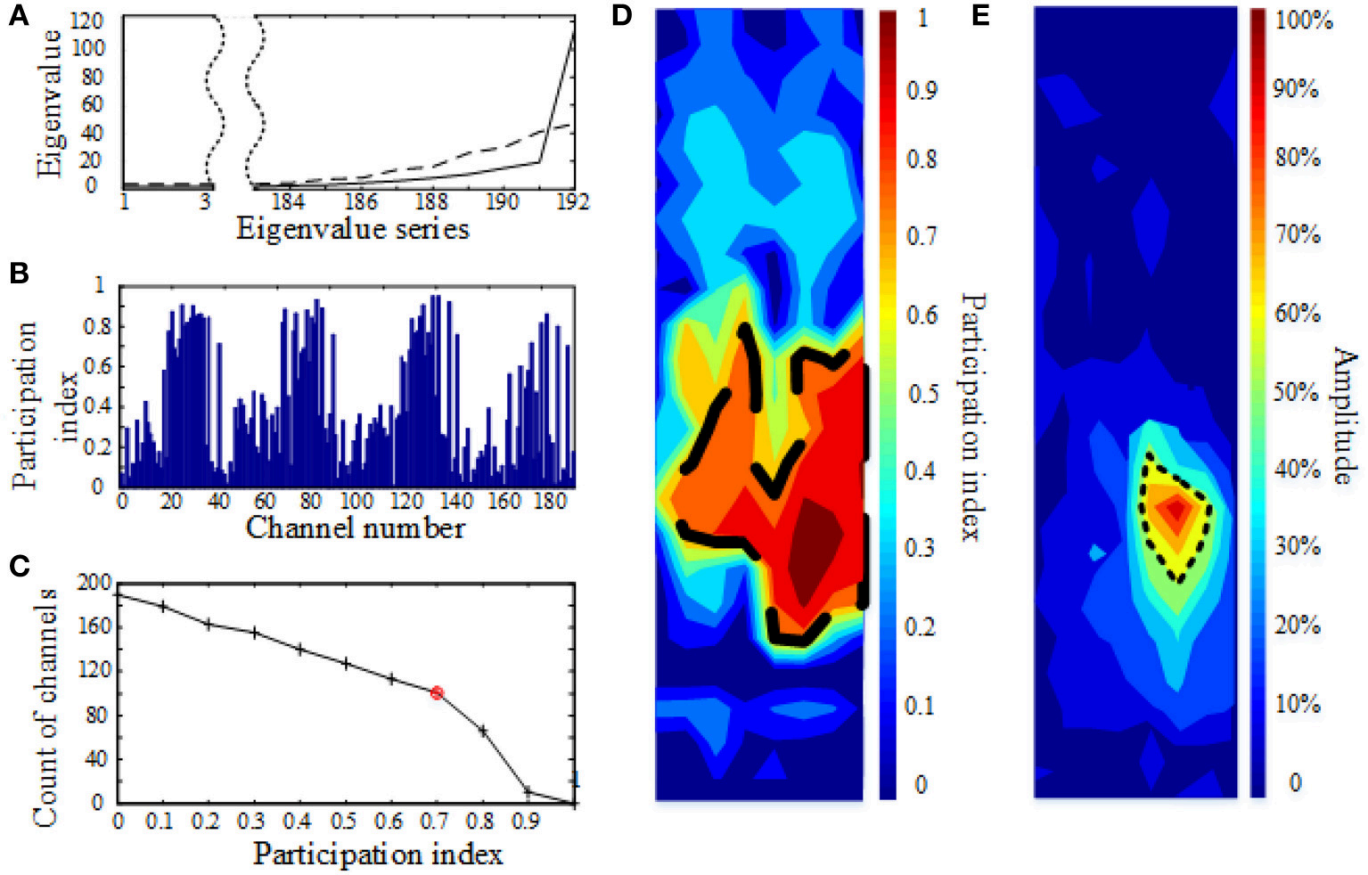
**Example of the proposed method in action (A–D) and topography calculated using traditional, amplitude-based analysis (E) in one normal rat**. **(A)** Sorted eigenvalues (solid curve) of the correlation matrix (192 × 192) from 192 channels. The dashed curve is the three-standard-deviation threshold based on the randomized surrogate data. Only one eigenvalue is above the threshold, indicating only one cluster (functional network) is present. **(B)** Participation index of all 192 channels in the network. **(C)** Histogram of the participation index. The red circle indicates the knee point used to demarcate the cortical boundary in **(D)**. **(D)** Topography of PI. The color represents the PI value. The dashed black line is the boundary drawn according the knee point value. The area within the dashed boundary is the functional brain network. **(E)** Traditional topography based on amplitude of SEPs in the same rat.

Figure 2 shows an example of the proposed analysis and detected synchronized network, as well as the traditional, amplitude-based topography in one normal rat. The solid curve in Figure 2A shows the sorted eigenvalues of the correlation matrix (192 × 192) from 192 channels (one eigenvalue per channel, in ascending order). The dashed curve indicates the three-standard-deviation threshold calculated from the surrogate method. As can be seen in Figure 2A, only one eigenvalue is above the threshold, which means only one functional network exists. Figure 2B depicts the participation index of all 192 channels in the sole detected network. The channels are listed by their number in on the x-axis (see Figure 1A for the numbering scheme), and the y-axis indicates participation index (PI) of each channel. The larger the PI, the greater the involvement in the network. Figure 2C shows a histogram of the participation index. As PI decreases, the amount of channels with that PI increases. The value of PI when the increase in the number of channels slows down is the knee point (red circle) of the graph. This PI value is then used as the criterion to demarcate the boundary of the functional network. Figure 2D shows the topography of PI calculated from the preceding analysis. The color indicates the PI value. The dashed black line is the boundary drawn according to the knee point value (red circle in Figure 2C). The area within the dashed boundary is the functional brain network detected by our proposed method. Figure 2E shows the topography based on traditional, amplitude-based processing of SEPs from the same rat. The area surrounded by the black dashed line indicates channels with SEP amplitude over 60% of the maximal amplitude. This 60% threshold was set because it results in the highest consistency, as measured by Hosp et al. (2008). The cortical representation detected via the traditional method is substantially smaller than the functional network seen in Figure 2D.

As indicated by the surrogate analysis on eigenvalues (e.g., dashed line criteria in Figure 2A), 10 out of the 12 normal rats (83.3%) have only one synchronized brain network. The remaining two normal rats (16.7%) have one strong and 1 weak network. The weak network can be neglected since it has fairly small synchronization strength (PI < 0.2). In the peripheral nerve injured group, all rats (100%) have only one synchronized brain network.

**Figure 3:**
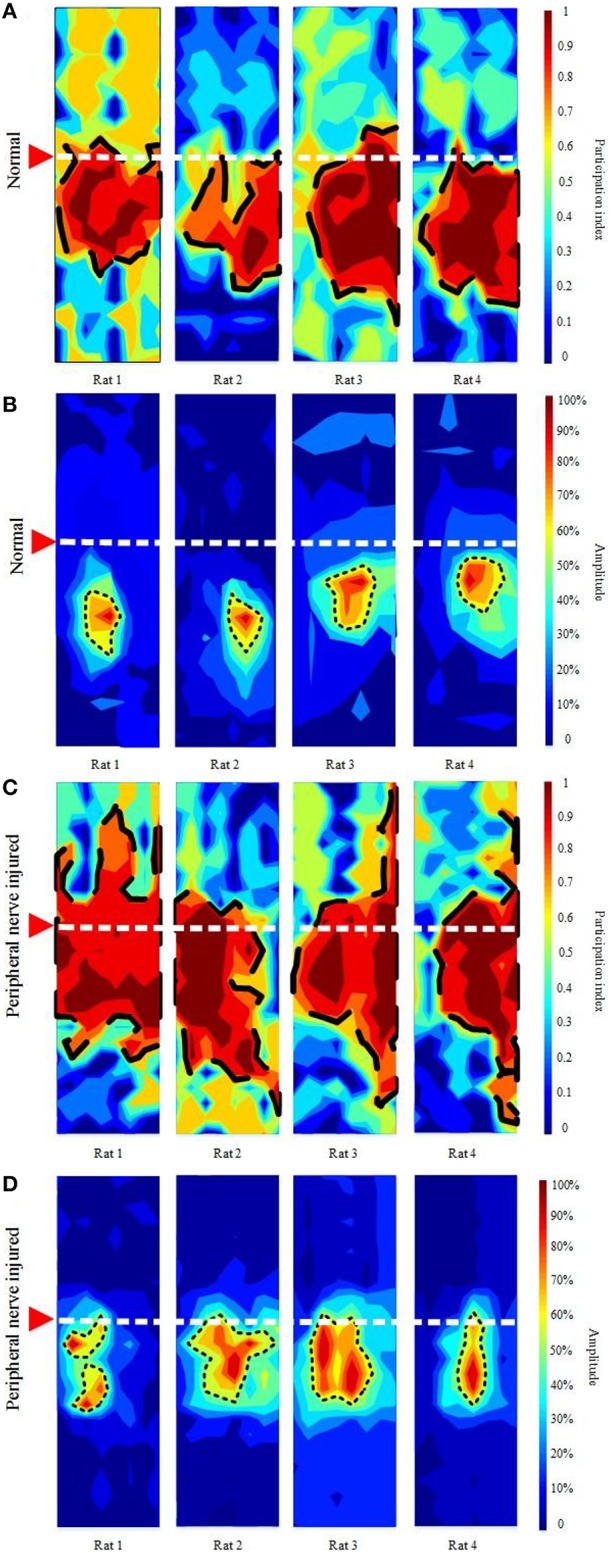
**Topographies of the cortical network calculated using the proposed method in the normal (A) and peripheral nerve injured (C) groups and traditional topography based on amplitude in the normal (B) and peripheral nerve injured (D) groups**. The rats shown in **(A)** are the same as those in **(B)**, and the rats in **(C)** are the same as those in **(D)**. The red solid arrow indicates the bregma. The dashed white line crosses the bregma and divides the cortex into anterior and posterior parts.

Figure 3 shows the topographies from the proposed and traditional methods in the normal and peripheral nerve injured rats. The red solid arrow indicates the bregma. The dashed white line crosses the bregma and divides the cortex into anterior and posterior parts. In the normal group (Figure 3A), all of the network shapes calculated from the proposed method appear regular, and the network areas are almost entirely posterior to the bregma. However, in the peripheral nerve injured group (Figure 3C) the network shapes appear more irregular and extend more anteriorly as well as posteriorly. The network area for the injured group is significantly larger (normal: 16.82 ± 3.93 mm^2^ vs. peripheral nerve injured: 21.69 ± 2.92 mm^2^, *P* < 0.05). There are considerable individual variations in the shape and location of the network after peripheral nerve injury, which is also seen in the traditional amplitude-based topography (Figures 3B,D).

## Discussion

The purpose of this study is to explore an objective and comprehensive method for somatotopy. The findings here demonstrate that the synchronized network determined from SEPs can reliably reveal somatosensory representation. However, further exploration is necessary.

Topography of SEPs based on amplitude and latency has been studied systematically (Hollenberg et al., 2006; Hosp et al., 2008). The advantages are clear: the approach integrates information from multiple sites and is easy to understand and apply. However, the drawbacks are that the peaks in the SEPs are analyzed alone and the analysis of the wave is somewhat subjective, therefore raising the need for further work.

The synchronized network calculation approach adopted in this study minimizes the interference of amplitude. Eigen analysis on the correlation matrix is used to calculate the synchrony strength among the channels. A random-phase surrogate data method is used to eliminate the bias effect and uncover independent networks (Li et al., 2007).

It is known that the cortical component of SEPs is near-field potential, and the cortical signals always localize to a very limited area in the cortex. In this study, 10 of the 12 normal rats (83.3%) and all of the four peripheral nerve injured rats (100%) show only one functional network. This result shows a the consistency between the traditional SEP area and the functional network. Interestingly, the functional network area is much larger than the traditional representation. The reason is probably because low-amplitude SEPs, which are numerous, are artificially neglected in traditional amplitude-based analysis. This study shows that the low-amplitude SEPs share high correlation with higher-amplitude SEPs and are important to understanding the cortical functional connectivity.

Previous studies have shown that the somatosensory representation of the rat is located on the convex side of the cerebral hemisphere, its shape is regular, and the location is relatively stable. Our results are consistent with the above. Cortical excitability and the amplitude of SEPs increase shortly after peripheral nerve or spinal cord injury (Han et al., 2013; Bazley et al., 2014). Our study found that after the peroneal nerve was cut, the amplitude of SEPs increased, network distribution expanded anteriorly and posteriorly, and the shape of the network became irregular. These findings reflect network plasticity after the peripheral nerve injury.

The mechanisms for the cortical excitation shortly after spinal or peripheral injury were investigated in several past studies (Calford, 2002; Pelled et al., 2007; Han et al., 2013). The central nervous system in adults is capable of post-trauma reorganization. First, inhibitory effects partially mediated by transcallosal projections and excitatory effects mainly mediated by thalamocortical projections shift the balance in short-term brain reorganization after peripheral nerve injury. With greater stimulus intensity for the injured sciatic nerve, inputs through the spared tibial nerve increase, thus thalamocortical inputs increase and inhibit transcallosal projections. Nevertheless, long-term plasticity after peripheral nerve injury and the balance of transcallosal projections and thalamocortical projections following injured sciatic nerve stimulation needs further exploration (Han et al., 2013). Second, loss of sensory inputs from the peroneal nerve immediately led to the expansion of spared tibial nerve representation, which is typical for intra-cortical plasticity (Calford, 2002; Pelled et al., 2007).

In conclusion, the determination of synchronized networks from SEPs can reveal functional brain networks. Compared to the results of traditional SEP-morphology-based analysis, the functional network area is far larger. Furthermore, our proposed method can also reveal the cortical plasticity which occurs quickly after the peripheral nerve is acutely injured.

## Author contributions

XQ is responsible for part of design of this study, animal experiment, part of data analysis and writing of the manuscript. JY is for part of data analysis. XL is for manuscript proofreading. PZ is for part of the idea of this study. XL is for the idea of this study and part of the design.

### Conflict of interest statement

The authors declare that the research was conducted in the absence of any commercial or financial relationships that could be construed as a potential conflict of interest.
